# Report and literature review of four cases of *EWSR1::NFATC2* round cell sarcoma

**DOI:** 10.1186/s13000-024-01443-y

**Published:** 2024-01-22

**Authors:** Lili Liu, Lan Li, Yi Ding, Fangzhou Kong, Wenfa Mo, Hongtao Ye, Danhua Shen

**Affiliations:** 1 Department of Pathology, Peking University People’s Hospital, Beijing, People’s Republic of China; 2 Department of Pathology, Beijing Jishuitan Hospital, The Fourth Medical College of Peking University, Beijing, People’s Republic of China; 3 Department of Pathology, Affiliated Hospital of Guilin Medical University, Guilin, People’s Republic of China; 4 Department of Histopathology, Royal National Orthopaedic Hospital NHS Trust, Brockley Hill, Stanmore, Middlesex HA7 4LP UK

**Keywords:** *NFATC2* sarcoma, Histopathological features, Immunohistochemistry, Molecular analysis

## Abstract

**Background:**

*EWSR1::NFATC2* rearranged sarcomas are a group of rare round, undifferentiated sarcomas with clinicopathological features different from those of Ewing's sarcoma (ES) family and other non-ES sarcomas. We report 4 cases of this rare sarcoma and review their features.

**Materials and methods:**

Four cases of *EWSR1::NFATC2* rearranged round cell sarcoma of the bone from the Pathology Department of Peking University People's Hospital were retrospectively studied. Clinical and pathological data were summarized, and immunohistochemical staining, fluorescence in situ hybridization (FISH), and Next-generation sequencing (NGS) were performed. Relevant literature reports were also reviewed.

**Results:**

Among the four cases of *EWSR1::NFATC2* rearranged round cell sarcoma, three were male, and one was female, with the age ranged from 14 to 34 years old at diagnosis (mean age: 27.5 years). All tumors were located in the femur and ranged in size from 4 to 8cm (mean 6cm), involving the surrounding soft tissues. All four patients underwent surgical treatment, and three received chemotherapy and radiotherapy postoperatively. Follow-up results showed that all four patients were alive. Histologically, the tumors exhibited small round cell sarcoma phenotype, with the stroma rich in mucin or exhibiting a glassy appearance. The tumor cells diffusely expressed CD99, NKX2.2, NKX3.1 and focal expression of CK and EMA was observed. FISH analysis showed that *EWSR1* gene rearrangement was detected in all 4 cases, accompanied by 5' locus amplification. EWSR1::NFATC2 fusion probe demonstrated multi yellow fusion signals. NGS identified *EWSR1::NFATC2* breakpoints in exon 9 and exon 3 in all 4 cases. The average follow-up duration of the study group was 88 months (range from 26—180 months). One case experienced both local recurrence and metastasis to the lung and chest wall. One case presented with local recurrence. The remaining two cases did not have the recurrence or metastasis.

**Conclusion:**

Although the disease can locally recur and metastasize to the lungs, its mortality rate is significantly lower than that of Ewing sarcoma and other high-grade small round cell undifferentiated sarcomas. Therefore, it supports to classify this tumor as a separate subtype of small round cell sarcoma.

## Background

Among bone and soft tissue small round cell sarcomas, the most common type is Ewing sarcoma, which is characterized by balanced translocation of the *EWSR1* gene on 22q12 as a molecular genetic feature. The fusion partners of this gene include *FLI-1* and *ERG*, accounting for 90% and 5%, respectively [1, 2]. In addition, *EWSR1* can also undergo translocation with some less common genes, namely *ETV1*, *ETV4*, and *FEV*. With the development of molecular techniques such as RNA sequencing, rare *EWSR1* fusion partners such as *SP3*, *PATZ1*, and *NFATC2* have been detected in some small round cell sarcomas [4–6]. In the fifth edition of the classification of soft tissue and bone tumors, these tumors are classified as *EWSR1*-*non*-ETS small round cell sarcomas [3]. These cases are very rare, and only a few reports about *EWSR1/FUS::NFATC2* fusion gene in small round cell sarcoma can be found in the literature [4–24]. This article reports four cases of *EWSR1::NFATC2* sarcoma, reviews the literature, and explores the clinical and pathological characteristics as well as molecular genetic features of this tumor.

## Materials and methods

### Case selection

Four cases of *EWSR1* gene rearrangement positive with red signal amplification (5’ of the *EWSR1* locus) undifferentiated small round cell sarcoma were selected. Cases 1—3 were from the Pathology Department of Peking University People's Hospital, and case 4 was a consultation case from Jishuitan Hospital. The diagnoses of all the cases were confirmed by senior bone and soft tissue pathologists in the Pathology Department of Peking University People's Hospital. Clinical and follow-up data were obtained from the clinical medical database and follow-up telephone calls.

### Histopathology and immunohistochemistry

The tissues were fixed with 4% neutral formaldehyde, embedded in paraffin, cut into 4μm thickness, and stained with routine hematoxylin–eosin (HE). EnVision two-step method was used for immunohistochemical detection. Antibodies used were CD99 (O13, 1:200, Beijing Jingqiao Yatu Biotechnology Co., Ltd, China.), SMA(1A4, 1:120, Beijing Jingqiao Yatu Biotechnology Co., Ltd, China.), Desmin (Ep15, 1:400, Beijing Jingqiao Yatu Biotechnology Co., Ltd, China.), Cytokeratins (AE1/3, 1:200, Fuzhou Maixin Biotechnology Development Co., Ltd, China.), S-100(15E2E2 + 4C4.9, 1:200, Beijing Zhongshan Jingqiao Biotechnology Co., Ltd, China.), Vimentin (EP21, 1:200, Beijing Zhongshan Jingqiao Biotechnology Co., Ltd, China.), EMA (E29, 1:200, Beijing Zhongshan Jingqiao Biotechnology Co., Ltd, China.), NKX2.2 (EP336, 1:200, Beijing Zhongshan Jingqiao Biotechnology Co., Ltd, China.), NKX3.1 (EP356, 1:100, Beijing Zhongshan Jingqiao Biotechnology Co., Ltd, China.) and MUC4 (8G7, 1:100, Beijing Zhongshan Jingqiao Biotechnology Co., Ltd, China.).

### Fluorescence in situ hybridization (FISH)

(1) Experimental method: FISH detection was performed using 4% neutral formalin-fixed, paraffin-embedded, 3μm thick white slides. *EWSR1* dual-color break-apart probe (Abbott Molecular, USA) and *EWSR1::NFATC2* dual-color fusion probe (Wuhan Kanglu Biotechnology Co., Ltd, China) were used. The experiment was performed according to the instructions provided with the kit. (2) Interpretation criteria: *EWSR1* break-apart probe: cells were observed under blue fluorescence, and isolated, intact and non-overlapping cell nuclei were selected for counting. Red and green signals were separated, and the distance between them was greater than twice the diameter of one signal. 100 cells were randomly counted, and if ≥ 10% of the cells showed separation signals, it was considered positive. *EWSR1::NFATC2* fusion probe: similarly, isolated, intact and non-overlapping cell nuclei were selected for counting, and both red and green signals were amplified, and the red and green signals fused together, showing yellow signal amplification. If ≥ 10% of cells showed this fusion pattern, it was judged as fusion-positive.

### Next-generation sequencing (NGS)

NGS analysis was performed on the patient's paraffin-embedded tumor samples. RNA was first extracted from FFPE samples using the RNA extraction kit from Qiagen, Germany. The total RNA concentration was measured using the Qubit RNA HS assay kit (ThermoFisher, catalog number: Q32852), and the quality control for integrity (DV200) was performed using the Agilent RNA 6000 Pico assay kit (Agilent, catalog number: 5067–1513). The detected total RNA amount was not less than 50 ng, with a DV200 value of not less than 30%. The extracted RNA was used for RNA library preparation. The starting amount of total RNA was at least 500 ng, and rRNA was removed using the NEBNext rRNA Depletion Kit (Human/Mouse/Rabbit) (NEB, catalog number: E6350). The RNA underwent fragmentation, primer and first-strand cDNA synthesis, second-strand synthesis, SPRI purification, end repair and A-tailing, and adapter ligation steps, followed by RNA sequencing using the Gene + Seq-2000 platform (GenePlus). The Gene + OncoBox (GenePlus) was used to identify fusion gene products. All steps were performed according to the instructions provided with the NEBNext Ultra II Directional RNA Library Prep Kit (NEB, catalog number: E7760).

## Results

### Clinical data

**Fig. 1:**
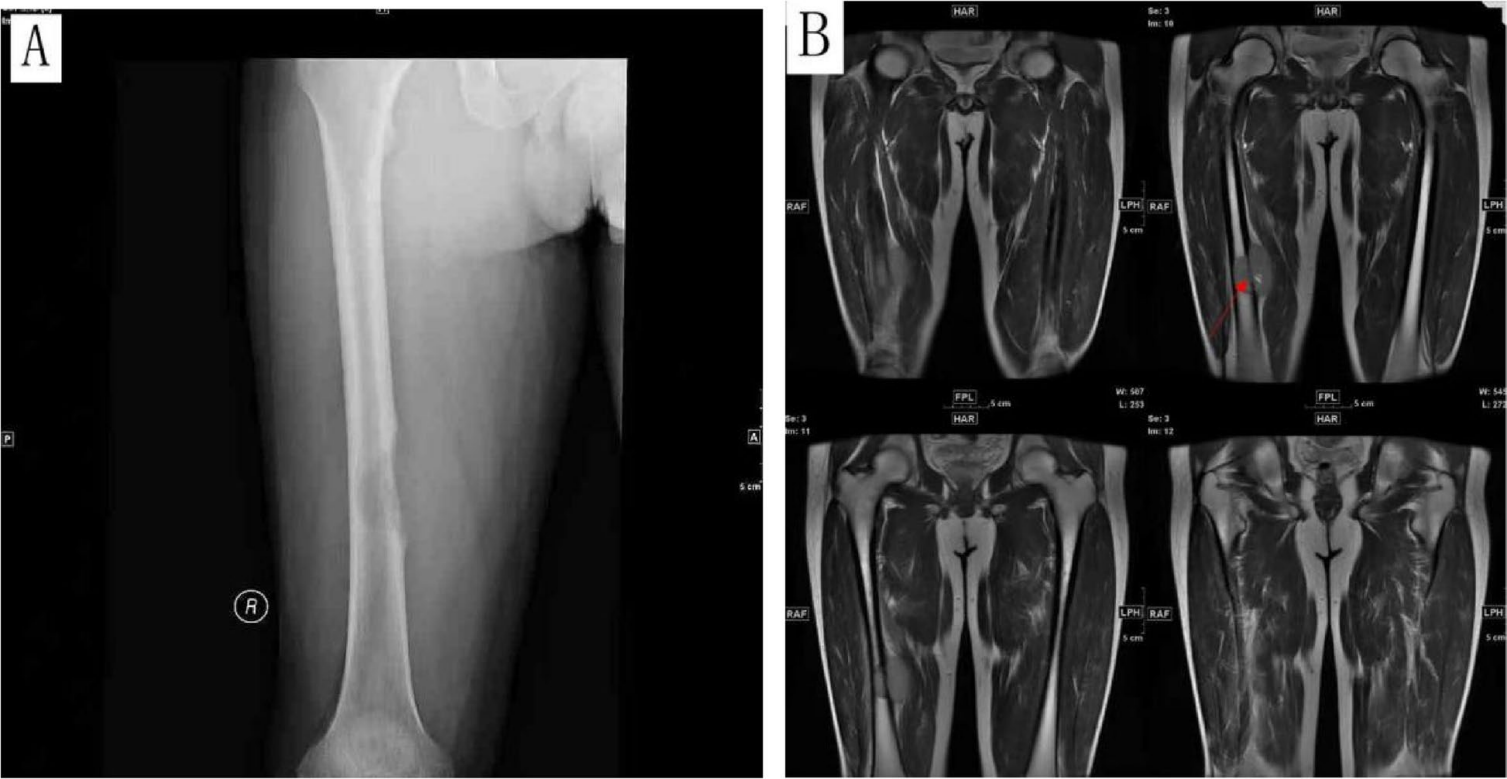
Case 1: Radiograph (**A**) and MRI (**B**) of the right femoral shaft tumor show lytic lesions involving the bone and surrounding soft tissues

Four cases, including 3 males and 1 female, with ages ranging from 14 to 34 years (average age 27.5 years). All 4 tumors occurred in the femur and the maximum size from 4 to 8 (mean 6 cm), with one case discovered by pathological fracture and the other 3 cases discovered due to painful masses. Imaging analyses showed that all lesions were osteolytic with indistinct borders on plain X-rays (case 1). Magnetic resonance imaging showed T2WI sequences. The lesion was located in the middle and lower femur, with local bone destruction, high signal and soft tissue mass protruding from the surrounding structure (Fig. 1A, B). Patients in cases 1 to 3 received Ewing's sarcoma chemotherapy regimen after surgical resection of the tumor, with case 2 also receiving radiation therapy. Case 4 underwent complete surgical resection only and did not receive further chemotherapy or radiation therapy. The follow-up period ranged from 26 to 180 months. The follow-up results showed that case 1 developed lung and chest wall metastasis 48 months after the initial diagnosis. Case 1 and case 2 occurred local recurrence at 23 months and 72 months after initial onset respectively, while the other 2 cases (case 3 and case 4) did not experience recurrence or metastasis. All 4 patients are currently alive. In addition to the 4 cases reported in this study, a total of 61 relevant studies and reports were reviewed, and the clinical characteristics are summarized in Table 1.

**Table 1:** Clinical and pathological features of *NFATC2*-rearranged sarcomas (including previous reported cases and cases from this study)

Study	Case	Age(y)/sex	Primary location	Local recurrence (mo)	Metastatic	Therapy	Outcome(months)/status
Current study	1	26/M	Femur	Yes(23)	Yes	Surgery + CT	84/AWD
	2	20/M	Femur	Yes(72)	No	nCT + Surgery + RT	180/AWD
	3	14/M	Femur	No	No	nCT + Surgery	26/AWOD
	4	32/F	Femur	No	No	Surgery	61/AWOD
Makise. [7]	5	26/F	Femur	Yes(24)	No	Surgery	NR
Perret^a^. [8]	6	27/M	Tibia	No	No	nCT + Surgery	102/AWOD
	7	66/F	Femur	Yes(4)	Yes	Surgery	32/DOD
	8	28/F	Tibia	No	No	nCT + Surgery	5/AWOD
	9	19/F	Ilium	-	No	nCT + RT	8/AWD
	10	58/M	Femur	-	No	nCT + Surgery	Recent case
Yoshida. [9]	11	39/M	Femur	NR	NR	NR	NR
	12	46/M	Femur	NR	NR	NR	NR
	13	27/M	Retroperitoneum	NR	NR	NR	NR
	14	31/F	Scapula	NR	NR	NR	NR
	15	36/M	Neck	NR	NR	NR	NR
	16	51/M	Forearm	NR	NR	NR	NR
	17	78/F	Tibia	NR	NR	NR	NR
	18	38/M	Thigh	NR	NR	NR	NR
	19	44/M	Thigh	NR	NR	NR	NR
	20	36/M	Fibula	NR	NR	NR	NR
	21	62/F	Femur	NR	NR	NR	NR
	22	49/M	Femur	NR	NR	NR	NR
Diza-Perez. [10]	23	28/M	Tibia	No	No	nCT + RT + Surgery	144/AWOD
	24	39/M	Femur	No	No	nCT + RT + Surgery	30/AWOD
	25	28/M	Humerus	No	No	nCT + RT + Surgery	Recent case
	26	46/M	Femur	Yes(5.9)	Yes	nCT + RT + Surgery	5.9/AWD
Wang. [11]	27	67/M	Radius	No	No	nCT + Surgery	14/AWOD
	28	32/M	Periclavicular	No	No	nCT + RT + Surgery	24/AWOD
	29	42/M	Radius	No	Yes	nCT + Surgery	16/AWD
	30	24/F	Gastrocnemius muscle	No	No	nCT + Surgery	23/AWOD
	31	42/M	Radius	No	Yes	nCT + Surgery	93/DOD
	32	59/M	Periclavicular	Yes(6)	No	nCT + RT + Surgery	144/AWOD
Koelsche^b^. [12]	33	51/M	Humerus	Yes(24)(*n* = 1)	Yes(*n* = 1)	nCT + Surgery	NA/AWOD(*n* = 1)
	34	56/F	Femur	No(*n* = 2)	No(*n* = 2)	(*n* = 3)	72/Alive(*n* = 1)
	35	17/F	Humerus				24/Alive(*n* = 1)
Mantilla. [13]	36	67/M	Thigh	NR	NR	Surgery	LOST
Bode-Lesniewska. [14]	37	34/F	Femur	NR	Yes	nCT + Surgery	132/AWOD
	38	42/M	Tibia	No	No	Surgery	102/AWOD
	39	60/F	Intra-abdominal	No	No	Surgery	8/AWOD
	40	12/M	Humerus	No	No	Surgery	8/AWOD
Yau. [15]	41	43/M	Femur	No	No	RT, nCT + Surgery	12/AWOD
Watson. [16]	42	49/F	Femur	NR	NR	NR	NR
	43	32/M	Humerus	NR	NR	NR	NR
	44	12/F	Tibia	NR	NR	NR	NR
	45	61/M	Calf	NR	NR	NR	NR
	46	23/M	Femur	NR	NR	NR	NR
	47	33/M	Femur	NR	NR	NR	NR
	48	43/M	Femur	NR	NR	NR	NR
Toki. [17]	49	NR	NR	NR	NR	NR	NR
Antonescu. [18]	50	42/M	Femur	NR	NR	NR	NR
Machado. [19]	51	NR	Fibula	NR	NR	NR	NR
Cohen. [20]	52	24/F	Calf	No	No	nCT + Surgery	12/AWOD
Brohl. [21]	53	15/M	Femur	NR	NR	NR	NR
Kinkor. [22]	54	12/M	Femur	No	No	nCT + Surgery	11/AWOD
	55	28/M	Humerus	Yes	Suspicious lung	Surgery + metastatic CT	53/AWD
Sadri. [23]	56	30/M	Femur	Yes(30)	No	Surgery + CT	36/AWOD
Romeo. [24]	57	32/M	Bone	NR	No	NR	64/AWOD
Szuhai. [6]	58	39/M	Humerus	NR	NR	NR	NR
	59	16/M	Femur	NR	NR	NR	NR
	60	21/M	Thigh soft tissue	NR	NR	NR	NR
	61	25/M	Femur	NR	NR	NR	NR

*AWOD* Alive without disease, *AWD *Alive with disease, *DOD* Dead of disease, *M* Male, *F* Female, *NR *Not reported, *nCT* Neoadjuvant chemotherapy, *CT* Chemotherapy

^a^Two cases from this series were originally reported in the study of Watson et al

^b^Two cases from this series were originally published in the study by Szuhai et al

### Histology and immunohistochemistry

**Fig. 2:**
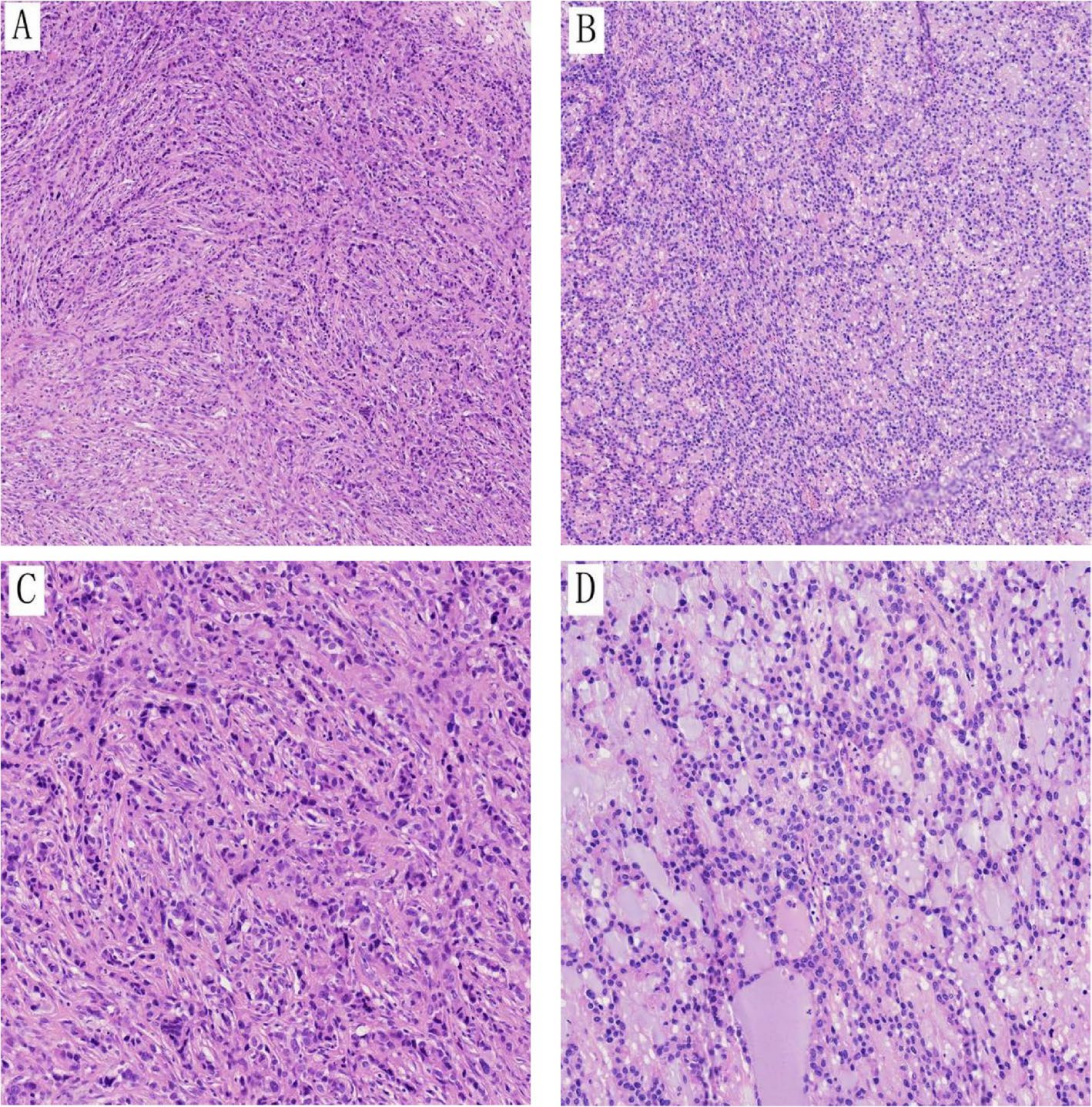
**A**, **B** Microscopic examination reveals the tumor with abundant fibrous or mucinous stroma (10x); hematoxylin and eosin (H/E) stain. **C**, **D** Tumor cells are arranged in small beams, cords, or clusters. At high magnification, tumor cells appear as monomorphic small round cells with eosinophilic and pale-staining cytoplasm, and inconspicuous or small nucleoli (20x); hematoxylin and eosin (H/E) stain

**Fig. 3:**
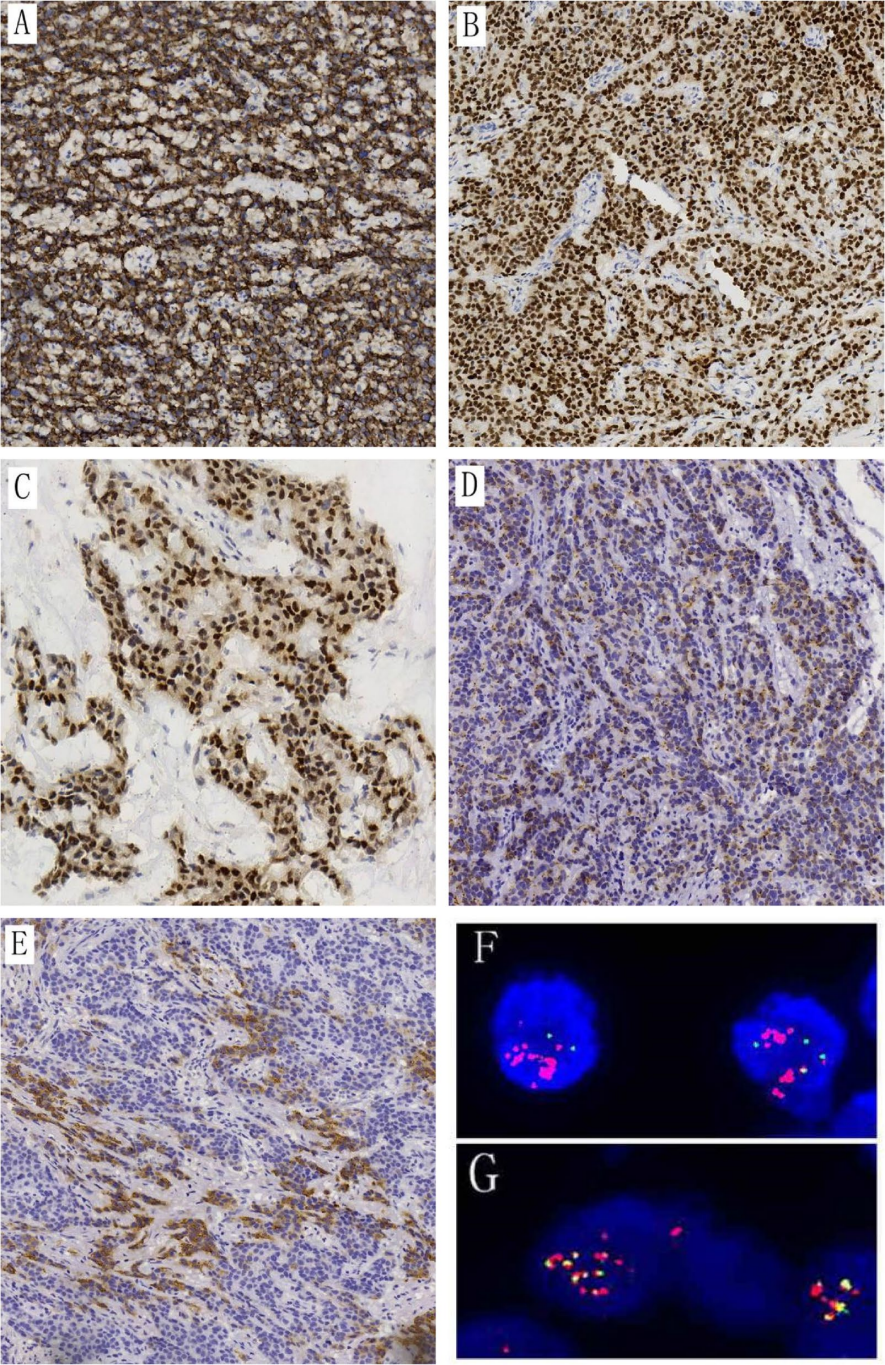
**A**, **B**, **C** Immunohistochemical staining shows diffuse expression of CD99 and NKX2.2 in all tumor cells, along with diffuse nuclear positivity for NKX3.1 (20x). **D**, **E** Two cases show focal expression of CK and EMA (20x). **F** FISH detection using EWSR1 probe shows red and green signals simultaneously, along with amplification of the 5' of the *EWSR1* locus (red signal). **G** FISH detection using *EWSR1::NFATC2* fusion probe shows *NFATC2* as the ligand gene for *EWSR1*, accompanied by amplification of fusion signals

Histologically, the tumor consisted of small to medium-sized round or oval cells arranged in nests, cords, trabeculae, or pseudoglandular patterns. The stroma contained abundant mucin and hyalinization fibers, resembling myoepithelioma of soft tissue (Fig. 2A, B). The cytoplasm was either eosinophilic or pale (Fig. 2C, D). Across all 4 cases, the mitotic activity per mm^2^ ranged from 4 to 20 (mean 10). Immunohistochemical results are shown in Table 2. CD99 showed varying degrees of membranous staining in all tumors (Fig. 3A). Our cases showed diffuse strong nuclear expression of NKX2.2 and NKX3.1 (Fig. 3B, C). Three cases showed focal expression of CK and EMA (Fig. 3D, E).

**Table 2:** Immunohistochemical results

Case	CK	EMA	CD99	NKX2.2	NKX3.1	S100	SMA	Desmin	MUC4
Case 1	focal +	focal +	+	+	+	-	-	-	-
Case 2	focal +	focal +	+	+	+	-	-	-	-
Case 3	-	+	focal +	+	+	-	-	-	-
Case 4	-	-	+	+	+	-	-	-	-

### Molecular genetics

**Fig. 4:**
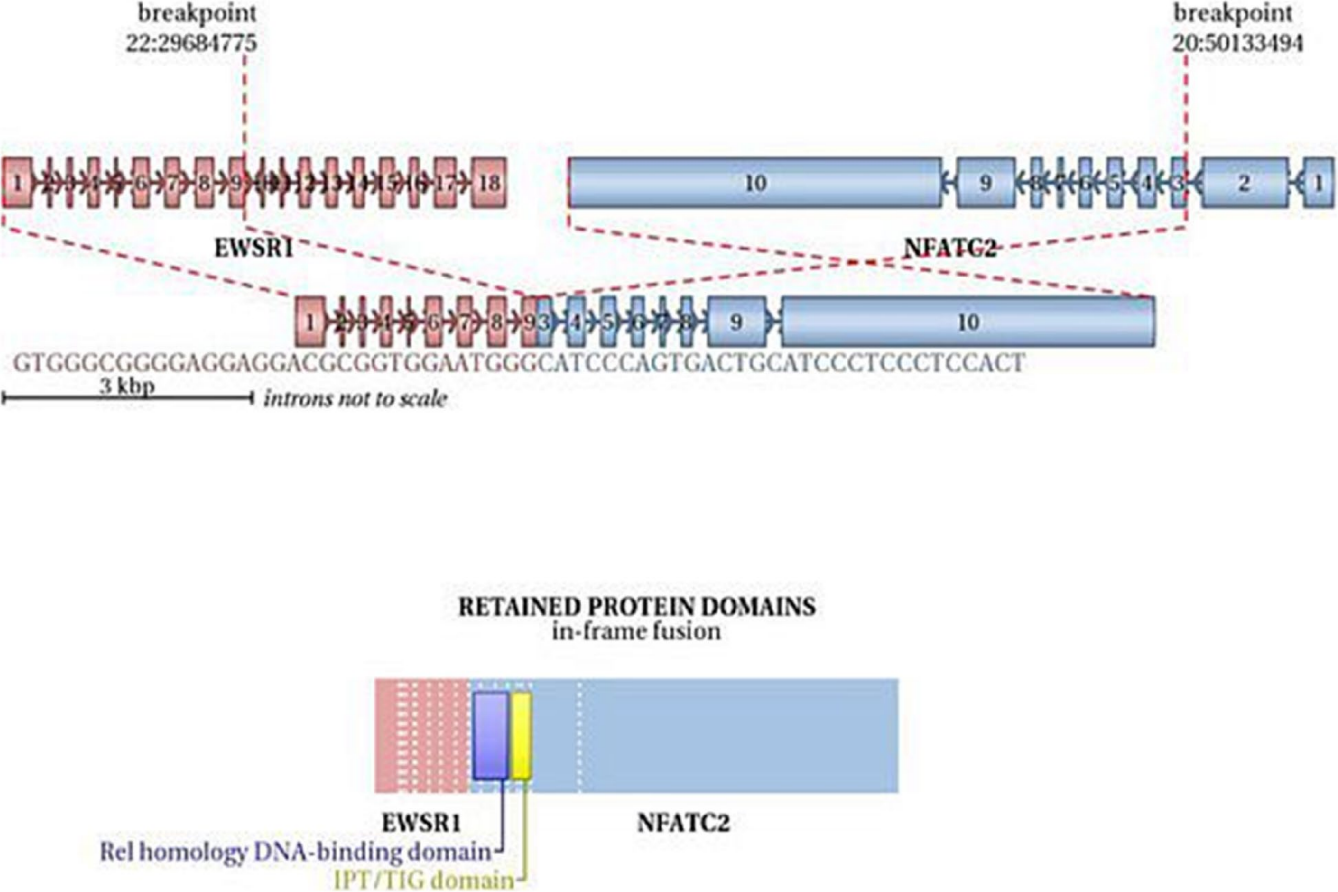
Exon 9 of the *EWSR1* gene and exon 3 of the *NFATC2* gene undergo. Breakpoints and fuse to form the *EWSR1::NFATC2*

In all four cases, FISH analysis using *EWSR1* break apart probe showed an unusual pattern for break-apart probes with one to two fused signals, one to two green signals and several grouped and amplified red signals, indicating the rearrangement of the *EWSR1* gene with the amplification of 5’ of the *EWSR1* locus (Fig. 3F). The percentages of positive cells in all 4 cases were from 52 to 90% by FISH detection. The *EWSR1::NFATC2* fusion probe showed multi yellow fusion signals, indicating *EWSR1::NFATC2* fusion gene (Fig. 3G). Next-generation sequencing identified the breakpoints of *EWSR1::NFATC2* fusion gene (Fig. 4).

## Discussion

The Ewing sarcoma family of tumors is a group of malignant mesenchymal neoplasms characterized by characteristic *EWSR1* gene rearrangements. They can occur in bone and soft tissue, and the pathological morphology is predominantly composed of small round cells. The fusion partners include *FLI1, ERG, SP3*, and others. In recent years, with the development of next-generation sequencing technology, some tumors associated with *EWSR1* gene rearrangements have shown unique histological and molecular genetic characteristics, such as *EWSR1/FUS::NFATC2* sarcoma. However, there is still controversy about whether to categorize it separately. *EWSR1::NFATC2* sarcoma is a rare type of sarcoma, first reported by Szuhai et al. [6] in 2009, and there have been 61 reported cases to date. This tumor exhibits specific clinical and histological features. In terms of clinical characteristics, *EWSR1/FUS::NFATC2* sarcoma mainly occurs in young males, with a male-to-female ratio of approximately 3:1. Long bones are the most common sites, with 26 cases occurring in the femur, 7 cases in the humerus, 6 cases in the tibia, and 3 cases in the radius. Patients often present with symptoms such as pain, swelling, and impaired mobility. The tumor has a rapid growth rate and can easily invade surrounding tissues and lymph nodes [25]. Therefore, when young individuals present with the aforementioned symptoms, the possibility of *EWSR1/FUS::NFATC2* sarcoma should be considered.

In terms of histology, *EWSR1/FUS::NFATC2* sarcoma typically presents as an undifferentiated small round cell sarcoma. The tumor cells are usually round or oval in shape, with finely granular chromatin, prominent nucleoli, and scant cytoplasm. The tumor cells are densely arranged, forming clusters or cords. Immunohistochemical staining shows that *EWSR1/FUS::NFATC2* sarcoma often expresses markers such as CD99, NKX3.1 [9]. Markers such as S-100 and Desmin are not expressed [8–10]. NKX2.2, which is a relatively specific marker for diagnosing Ewing sarcoma, can also be expressed in *EWSR1/FUS::NFATC2* sarcoma [11]. Therefore, histological and immunohistochemical examinations can provide important references for the diagnosis of *EWSR1/FUS::NFATC2* sarcoma.

FISH and NGS play an important role in the diagnosis of *EWSR1/FUS::NFATC2* sarcoma. Unlike classical Ewing sarcoma, this tumor exhibits a characteristic FISH pattern with not only the separation signal of *EWSR1* but also a clear amplification at the 5' of the *EWSR1* locus. This suggests the possibility of *EWSR1/FUS::NFATC2* sarcoma and can be validated using *EWSR1::NFATC2* or *FUS::NFATC2* fusion probe and NGS. Although *EWSR1/FUS::NFATC2* sarcoma has its unique FISH presentation, due to its rarity and non-exclusive molecular genetic features, the diagnosis of this tumor must be combined with histology and immunohistochemistry. For certain situations, such as *EWSR1::POU5F1* sarcoma and rare myoepithelial sarcoma, validation using *EWSR1/FUS::NFATC2* fusion probe is necessary. Additionally, the *EWSR1/FUS::NFATC2* gene rearrangement feature can also be detected in simple bone cysts [26], but it does not harbour the amplification of the fusion gene and exhibits different clinical manifestations, histology, and prognosis compared to *EWSR1/FUS::NFATC2* sarcoma. Therefore, although *EWSR1/FUS::NFATC2* sarcoma has a relatively specific FISH presentation, when diagnosing tumors with *EWSR1/FUS::NFATC2* gene rearrangement, it is necessary to consider factors such as the clinical presentation, histological changes, treatment and prognosis. In our review of the four cases reported in this study and previously published cases, almost all reported cases of *EWSR1::NFATC2* sarcoma exhibited the FISH pattern with separation of red and green signals accompanied by 5' amplification of the *EWSR1* locus. However, other tumors such as *EWSR1::POU5F1* sarcoma and rare myoepithelial sarcoma may also show separation with amplification signals in FISH testing, but they are accompanied by amplification at the 3' end or other locations [27]. Therefore, differentiation between these tumors can be achieved by combining clinical manifestations, histology, and various molecular testing methods for validation.

The most important differential diagnosis for *EWSR1/FUS::NFATC2* sarcoma is classical Ewing's sarcoma. Morphologically, these two tumors exhibit similar pathological features and are both small round cell tumors. However, *EWSR1/FUS::NFATC2* sarcoma cells form small fascicular or cord-like structures, with an interstitium that is rich in mucin or undergoes collagen degeneration. Additionally, this tumor also needs to be distinguished from the less common sclerosing epithelioid fibrosarcoma. In our case selection, we encountered a challenging case: FISH analysis detected *EWSR1* gene rearrangement, showing separation of red and green signals with 5' amplification, thus being included in this study. However, when verification was performed using the *EWSR1::NFATC2* fusion probe, the result was negative. Subsequent NGS confirmed the presence of *EWSR1::CREB3L1* rearrangement, and on top of this result, we performed additional immunohistochemical staining for MUC4, which showed positive expression. Considering the combined results of pathological morphology, immunohistochemistry, and molecular changes, the final diagnosis for this case was sclerosing epithelioid fibrosarcoma. Therefore, when FISH analysis shows separation of red and green signals with 5' amplification in *EWSR1* gene rearrangement, the possibility of sclerosing epithelioid fibrosarcoma should also be considered, and differential diagnosis can be made based on indicators such as positive expression of MUC4 and positive rearrangement of *EWSR1::CREB3L1* fusion probe in FISH analysis [28]. Due to the focal expression of epithelial markers such as CK and EMA in some cases of *EWSR1/FUS::NFATC2* sarcoma, combined with morphological features, it can be easily misdiagnosed as an myoepithelioma [29]. Furthermore, approximately 50% of myoepitheliomas have translocations involving the *EWSR1* gene, such as *EWSR1::POU5F1* sarcoma, and myoepithelioma can also exhibit positive expression of CD99 and NKX3.1, posing a significant diagnostic challenge. Immunohistochemical staining for NKX2.2 is helpful in distinguishing between the two, as myoepithelioma shows negative or focal weak positive expression of NKX2.2 [11]. In addition, extraskeletal myxoid chondrosarcoma (EMC) needs to be differentiated from *EWSR1/FUS::NFATC2* sarcoma. EMC typically occurs in soft tissues outside the bone, but there have also been reports of cases occurring in bone [30, 31]. The clinical presentation, histology, and immunohistochemical marker expression of bone EMC are similar to those occurring in deep soft tissues. Morphologically, EMC exhibits a round or slightly spindle-shaped arrangement of tumor cells forming cord-like structures, distributed in myxoid stroma, with large nuclei and eosinophilic cytoplasm. Its molecular hallmark is rearrangement of the *NR4A3* gene, with the most common fusion partner being *EWSR1.* Based on its molecular features, it can be distinguished from *EWSR1/FUS::NFATC2* sarcoma. In summary, the differential diagnosis of *EWSR1/FUS::NFATC2* sarcoma is complex with many pitfalls. Diagnosis cannot rely solely on FISH analysis. Even when FISH analysis shows positive separation of *EWSR1* gene, amplification, further verification with specific fusion probes is still necessary. If conditions permit, additional NGS testing should be conducted, and a comprehensive diagnosis should be made based on the combination of morphological features, immunohistochemistry, and molecular analysis.

In terms of prognosis, *EWSR1/FUS::NFATC2* sarcoma typically requires treatment such as surgical resection, radiation therapy, and chemotherapy. Some studies have shown that the prognosis of *EWSR1/FUS::NFATC2* sarcoma is related to factors such as age, tumor size, lymph node metastasis, and surgical resection. However, due to the rarity and heterogeneity of this tumor, assessing prognosis remains challenging. In our four cases, three underwent postoperative chemotherapy while one did not receive further treatment after surgery. The follow-up period ranged from 26 to 180 months, and two cases experienced local recurrence and lung metastasis, while the other two did not show recurrence or metastasis. Among the 30 cases with follow-up results, including the four cases reported in our study, the mortality rate of *EWSR1/FUS::NFATC2* sarcoma is not high, even though it can exhibit local recurrence and lung metastasis. Whether adjuvant chemotherapy can achieve good clinical results is still controversial, and more clinical cases need to be further studied. The latest research findings and novel treatment approaches for *EWSR1/FUS::NFATC2* sarcoma involve molecular pathology research, immunotherapy, targeted therapy, and gene-editing techniques. These research results and novel treatment approaches provide new insights and directions for the treatment and management of *EWSR1/FUS::NFATC2* sarcoma. The fusion breakpoints and fusion types in *EWSR1/FUS::NFATC2* sarcoma may impact the tumor's biological behavior and prognosis. *FUS::NFATC2* sarcoma has a worse prognosis than *EWSR1::NFATC2* sarcoma [8]. In recent years, immunotherapy has made significant progress in the field of cancer treatment, including some studies on soft tissue sarcoma, such as round cell sarcoma. Studies by Seligson et al. have shown that *EWSR1::NFATC2* sarcoma has a lower response to Ewing sarcoma-specific chemotherapy and suggest that drugs targeting the mTOR pathway may become targeted therapies for treating this disease [32].

## Conclusion

In terms of clinical, radiological, histological, and FISH detection findings, *EWSR1::NFATC2* fusion sarcoma exhibits distinct histopathological and molecular genetic features. Its clinical prognosis is different from classical Ewing sarcoma.

## Data Availability

No datasets were generated or analysed during the current study.

## Abbreviations

FISH: Fluorescence in situ hybridization
NGS: Next-generation sequencing
EMC: Extraskeletal myxoid chondrosarcoma
FFPE: Formalin-fixed, paraffin-embedded
